# Total contact casts versus removable offloading interventions for the treatment of diabetic foot ulcers: a systematic review and meta-analysis

**DOI:** 10.3389/fendo.2023.1234761

**Published:** 2023-09-26

**Authors:** Bin Li, Aifang Lin, Jianping Huang, Jianying Xie, Quanyong Liu, Chenxi Yang, Zhengmao Zhang

**Affiliations:** Department of Orthopedics, Yuhuan People’s Hospital, Taizhou, Zhejiang, China

**Keywords:** diabetic foot ulcers, total contact casts, removable offloading intervention, systematic review, meta - analysis

## Abstract

**Objective:**

This study aimed to evaluate the effectiveness of total contact casts (TCCs) versus removable offloading interventions among patients with diabetic foot ulcers (DFUs).

**Methods:**

A comprehensive search was done in databases Embase, Cochrane Library, and, PubMed. The references of retrieved articles were reviewed, up until February 2023. Controlled trials comparing the effects of TCCs with removable offloading interventions (removable walking casts and footwear) in patients with DFUs were eligible for review.

**Results:**

Twelve studies were included in the meta-analysis, involving 591 patients with DFUs. Among them, 269 patients were in the intervention group (TCC), and 322 in the control group (removable walking casts/footwear). The analysis revealed that the TCC group had higher healing rates (Risk Ratio(RR)=1.22; 95% confidence interval(CI):1.11 to 1.34, p<0.001), shorter healing time (Standard Mean Difference(SMD)=-0.57; 95%CI: -1.01 to -0.13, P=0.010), and elevated occurrence of device-related complications (RR=1.70; 95%CI:1.01 to 2.88, P=0.047), compared with the control group. Subgroup analysis illustrated patients using TCCs had higher healing rates than those using removable walking casts (RR=1.20; 95%CI:1.08 to 1.34, p=0.001) and footwear (RR=1.25; 95%CI:1.04 to 1.51, p=0.019), but they required comparable time for ulcer healing compared with those using removable walking casts (SMD=-0.60; 95%CI: -1.22 to 0.02, P=0.058) or footwear group (SMD=-0.52; 95%CI: -1.17 to 0.12, P=0.110). Although patients using TCCs had significantly higher incidence of device-related complications than those using footwear (RR=4.81; 95%CI:1.30 to 17.74, p=0.018), they had similar one compared with those using the removable walking casts (RR=1.27; 95%CI:0.70 to 2.29, p=0.438).

**Conclusion:**

The use of TCCs in patients with DFUs resulted in improved rates of ulcer healing and shorter healing time compared to removable walking casts and footwear. However, it is important to note that TCCs were found to be associated with increased prevalence of complications.

## Introduction

In 2021, around 536.6 million individuals aged 20 to 79 years had diabetes worldwide, and it is projected to reach 783.2 million by 2046 (1). Additionally, there are around 541 million people with abnormal glucose tolerance (2). The International Diabetes Foundation (IDF) reports that the number of people affected by diabetic foot ulcers (DFUs) has significantly increased from an estimated 9 to 26 million in 2015 to between 40 and 60 million worldwide (3). Among people with diabetes, foot ulcers develop in approximately 19% to 34% of cases. And once they occur, there is a high recurrence rate within 3-5 years (65%). Moreover, DFUs result in a 20% incidence of lifetime lower limb amputation, with a 5-year mortality rate after amputation ranging from 50% to 70% (4).

The global costs of diabetes care, including direct and indirect expenses, have substantially risen in recent years, primarily due to foot complications. In the United States alone, the annual direct cost of diabetes care is estimated at $273 billion, with an additional $90 billion in indirect costs. Complications arising from diabetic foot conditions result in increased hospital admissions, visits to the emergency department, outpatient visits, and utilization of home health care services, leading to yearly excessive expenditure that exceeds the standard cost of diabetes-related care by 50% to 200% (5, 6).

Neuropathic foot ulcers arise due to multiple factors, such as peripheral neuropathy, peripheral arterial disease, and structural deformities in the foot. However, elevated plantar pressure plays a crucial role in initiating neuropathic foot ulcers, particularly in the absence of protective sensation (4, 7). Moreover, effective unloading of the affected area is crucial for timely wound healing, as the presence of a neuropathic ulcer can cause significant delays. Therefore, offloading therapy is considered a vital treatment approach (8, 9).

Total contact casts (TCCs) were first introduced in 1984 for the treatment of plantar ulcers (10). As the first knee-high non-removable device, it became the standard method for offloading DFUs in the early stages (11). However, emerging evidence suggests that removable devices, such as cast walkers and therapeutic shoes, are equally effective compared to TCC (12–14). Only a limited number of quantitative analyses have been performed to compare TCC with other offloading interventions (15). Despite the existence of published meta-analysis comparing TCCs and removable offloading devices (15, 16), they focused on healing time and healing rate. There is no meta-analysis examined the effectiveness of these treatment options regarding healing time, healing rate, and device-related complications in patients with DFUs so far.

Therefore, this meta-analysis was conducted to quantitatively compare TCC with other offloading measures, such as removable walking casts and footwear, with respect to healing rate, healing time, and device-related complications in patients with DFUs. This study may shed light on the selection of appropriate offloading interventions to optimize treatment outcomes and minimize complications.

## Materials and methods

This study was conducted following the Cochrane Handbook for Systematic Reviews of Interventions (version 6.3) guidelines (17). The Preferred Reporting Items for Systematic Reviews and Meta-Analysis (PRISMA) statement served as its foundation (18).

### Search strategy

We searched databases including Embase, Cochrane Library, and PubMed until February 2023 to identify relevant studies that examined the outcomes associated with the use of TCCs versus removable offloading devices in patients with DFUs. Two reviewers (Bin Li and Zhengmao Zhang) conducted the literature search independently, and no restrictions were placed on language. The review team developed and piloted search strategies for databases of bibliographic records and clinical trial registries. Medical subject headings or Entree and text words like “diabetic foot” and “cast” were used. We also reviewed the reference lists of full-text articles. Detailed search strategies were seen in the **Appendix**.

### Inclusion criteria

Participants from various countries who were over 18 years old had neuropathic DFUs. The intervention involved using TCCs on the patients. The comparators were patients with DFUs who used removable offloading devices, including removable walking casts (knee-high and ankle-high removable devices) and footwear. Outcomes were ulcer healing rates, ulcer healing time, and device-related complications. Type of study design was randomized controlled trial or non-randomized controlled trial. Trials published in English, Dutch, Spanish, Italian, German, or Portuguese were considered for inclusion. The inclusion criteria did not impose restrictions based on the duration of reported DFUs, publication status, reported outcomes, or the outcome assessment instruments used.

The definition of Device-related Complications: Device-related Complications were defined as Complications which induced by device, including device failure, skin maceration or abrasions, infection.

### Exclusion criteria

Studies were excluded if (1) data were unavailable (2); they were conference summaries, animal experiments, case reports, and systematic reviews or meta-analyses (3); they were duplicate publications (4); their full text was unavailable.

### Study selection and data extraction

The review team members independently reviewed articles retrieved from databases. Firstly, the titles and abstracts of articles were reviewed to remove duplicate publications. The remaining articles then underwent full-text screening to identify studies satisfying the predefined inclusion criteria. In case of disagreements, discussions (Bin Li, Zhengmao Zhang, Jianying Xie, Quanyong LIu, and Chenxi Yang) took place, or, a third independent reviewer (Aifang Lin or Jianping Huang) was consulted, if necessary. The following information was extracted from individual studies: the first author’s name, publication year, country, participant characteristics, duration, outcome measures, and details of offloading interventions. Primary outcomes were healing rates, healing time, and incidence of device-related complications.

### Quality assessment

Risk of bias in individual RCTs was evaluated using the tool recommended by the Cochrane Reviews. This was conducted using the software Review Manager 5.4. Domains through which bias may be introduced into the results of RCTs included bias arising from the randomization process, bias in blinding participants and personnel, bias arising from measurement of outcomes, bias caused by missing outcome data, bias arising from selection of the reported results, and other sources of bias. In this process, any disagreements were resolve through discussion until a consensus was reached. For non-RCTs, quality assessment was done using the Newcastle–Ottawa Scale (NOS), whereas it was conducted using specific tools for cohort and case-control studies. A study can be awarded a maximum of 9 points, in which a maximum of 4, 2, and 3 can be given for the Selection, Comparability, and Outcome category, respectively. In our analysis, studies that scored above the median stars were considered to have relatively high quality, while those scoring below were deemed to have low quality.

### Statistical analysis

Analysis of parallel studies was performed, where the mean change from baseline and corresponding standard deviations (SDs) were calculated. In cases where SDs change were not provided, they were estimated using the SDs at baseline and endpoint. Meta-analysis was carried out, where the Mantel-Haenszel statistical method was used to produce standard mean differences (SMD), risk ratios (RRs), and 95% confidence intervals (CIs). We compared continuous outcomes between groups using standard mean differences (SMD) and 95% CIs, while differences between dichotomous outcomes were compared using relative risks (RRs) and 95% CIs. If no events were observed in one comparison group, we added 0.5 to both groups. As recommended by the Cochrane Handbook (17), trials with no outcome events in both arms were excluded from the meta-analysis when calculating RRs. A fixed-effects model was utilized for data analysis if p-value for heterogeneity was greater than 0.1, whereas a random-effects model was employed when it was 0.1 or lower. The chi-square test was done to assess heterogeneity, where *I* ^2^ > 50% indicated significant heterogeneity. We evaluated the significance of subgroup differences to determine how categorical confounding factors affected the outcome. Sensitivity analysis was done by omitting one trial at a time to assess the stability of the results of data analysis. Additionally, Egger’s test was conducted to evaluate publication bias when five or more trials of interest were analyzed. A p-value of less than 0.05 was considered the threshold of statistical significance. All the aforementioned analyses were performed using Stata/SE 15.0 software (Stata Corporation, College Station, TX).

## Results

**Figure 1:**
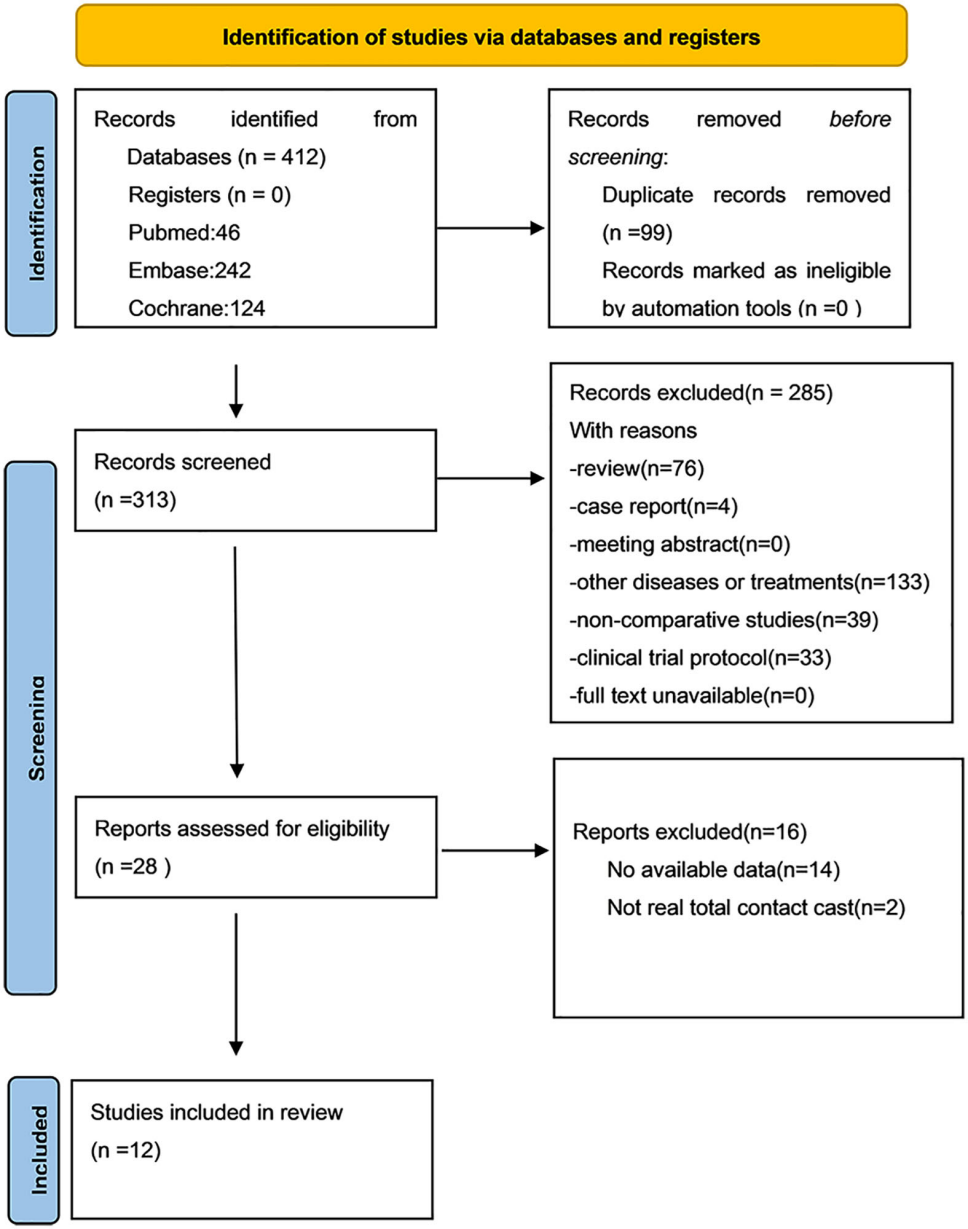
flow diagram.

In total, 412 articles were obtained from and Cochrane Library (n=124), Embase (n=242), and PubMed (n=46). Review of these articles produced 12 studies that were finally included in the present study (12–14, 19–27). **Figure 1** shows the literature selection process.

### Study characteristics

In total, 591 participants were involved in the 12 studies published between 2000 to 2016. Among them, 269 participants were in the intervention group (TCC) and 322 in the control group (removable walking casts/footwear). Specifically, removable walking casts were used as offloading devices in eight studies and footwear was utilized in four studies. The length of treatments spanned from one month to six months. Study characteristics are shown in **Table 1**.

**Table 1:** Characteristics of the eligible studies.

Study	Year	Country	participant	Sample size		Gende (M/F)	Age (year)		Intervention			Follow-up	Outcome
				CG	EG		CG	EG	CG		EG		
Piaggesi (12)	2016	Italy	Diabetic forefoot plantar ulcer	20	20	23/17	62.3 ± 9.2	61.4 ± 9.7	removable walking boot	Removable walking cast	Total contact casting	90d or up to complete re-epithelization	Healing rates Healing time Ulcer size reduction
Lavery (19)	2014	USA	Diabetic plantar ulcers	27	23	29/21	NA	NA	a removable boot with a shear-reducing foot bed	Removable walking cast	Total contact casting	Not Mentioned	Healing rates Healing time Shear-reducing foot bed
Strakhova (21)	2014	Russia	Neuropathic Diabetic forefoot plantar ulcer	20	20	21/19	54.1 ± 9.9	49.3 ± 12.0	ankle-foot pneumoorthosis with a HAS-337 TM Orlett	Removable walking cast	Total contact casting	6 months	Healing time
Gutekunst (20)	2011	USA	Neuropathic diabetic plantar ulcers	12	11	19/4	53 ± 10	55 ± 13	removable cast walker boot	Removable walking cast	Total contact casting	Not Mentioned	Healing time Pressure reduction
Faglia (13)	2010	Italy	Neuropathic diabetic plantar ulcers	22	23	30/15	61.7 ± 10.4	59.0 ± 8.5	removable cast walker	Removable walking cast	Total contact casting	90d	Ulcer surface reduction Healing time Complications Healing rates costs
Vandeweg (22)	2008	Netherlands	Neuropathic diabetic plantar ulcers	20	23	33/9	58.1 ± 11.1	64.8 ± 10.8	custom-made temporary footwear	Foot wear	Total contact casting	16w	Healing rates Healing time Complications Ulcer surface reduction
Caravaggi (23)	2007	Italy	Neuropathic diabetic plantar ulcers	29	29	NA	NA	NA	Aircast Pneumatic Walker	Removable walking cast	Total contact casting	90d	Healing rates Complications
Piaggesi (14)	2007	Italy	Neuropathic diabetic plantar ulcers	20	20	NA	61.1 ± 6.4	59.8 ± 8.2	Optima Diab walker	Removable walking cast	Total contact casting	12w	Complications Healing rates Healing time Time for placement Costs satisfaction
Van (24)	2003	France	diabetic plantar ulcers.	51	42	78/15	62 ± 7	58 ± 11	off-loading shoes	Foot wear	nonremovable fiberglass cast boot	Not Mentioned	Healing time complications
Birke (25)	2002	USA	neuropathic forefoot ulceration	57	13	NA	58.2 ± 11.5	47.3 ± 9.1	Healing shoe	Foot wear	Total contact casting	12w	Healing time Healing rates
Armstrong (26)	2001	USA	neuropathic foot ulcerations	20	19	32/7	NA	NA	removable cast walkers	Removable walking cast	Total contact casting	12w	Healing rates Healing time Activity of the patients
Caravaggi (27)	2000	Italy	Neuropathic diabetic plantar ulcers	24	26	34/16	59.2 ± 9.9	60.5 ± 10.7	a cloth shoe with a rigid sole with unloading alkaform insoles	Foot wear	Total contact casting	30d	Healing rates

### Study quality

**Figure 2:**
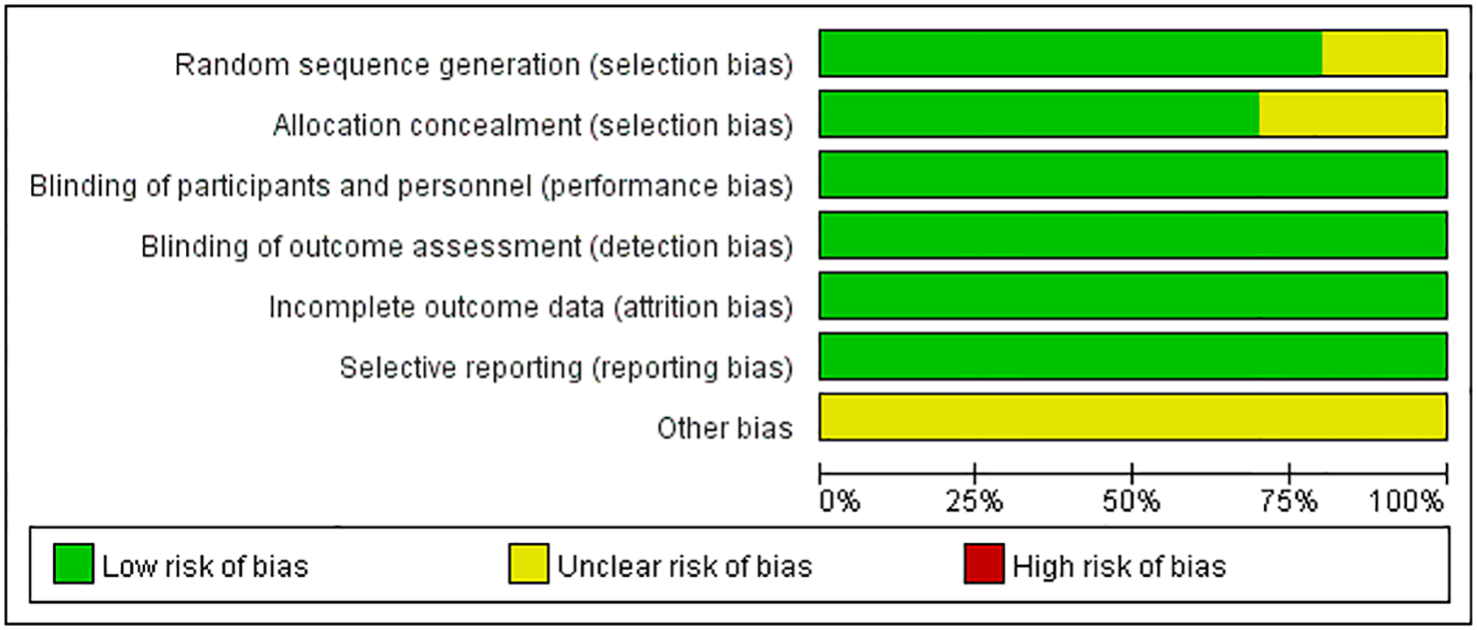
Risk of bias summary of RCTs.

**Figure 3:**
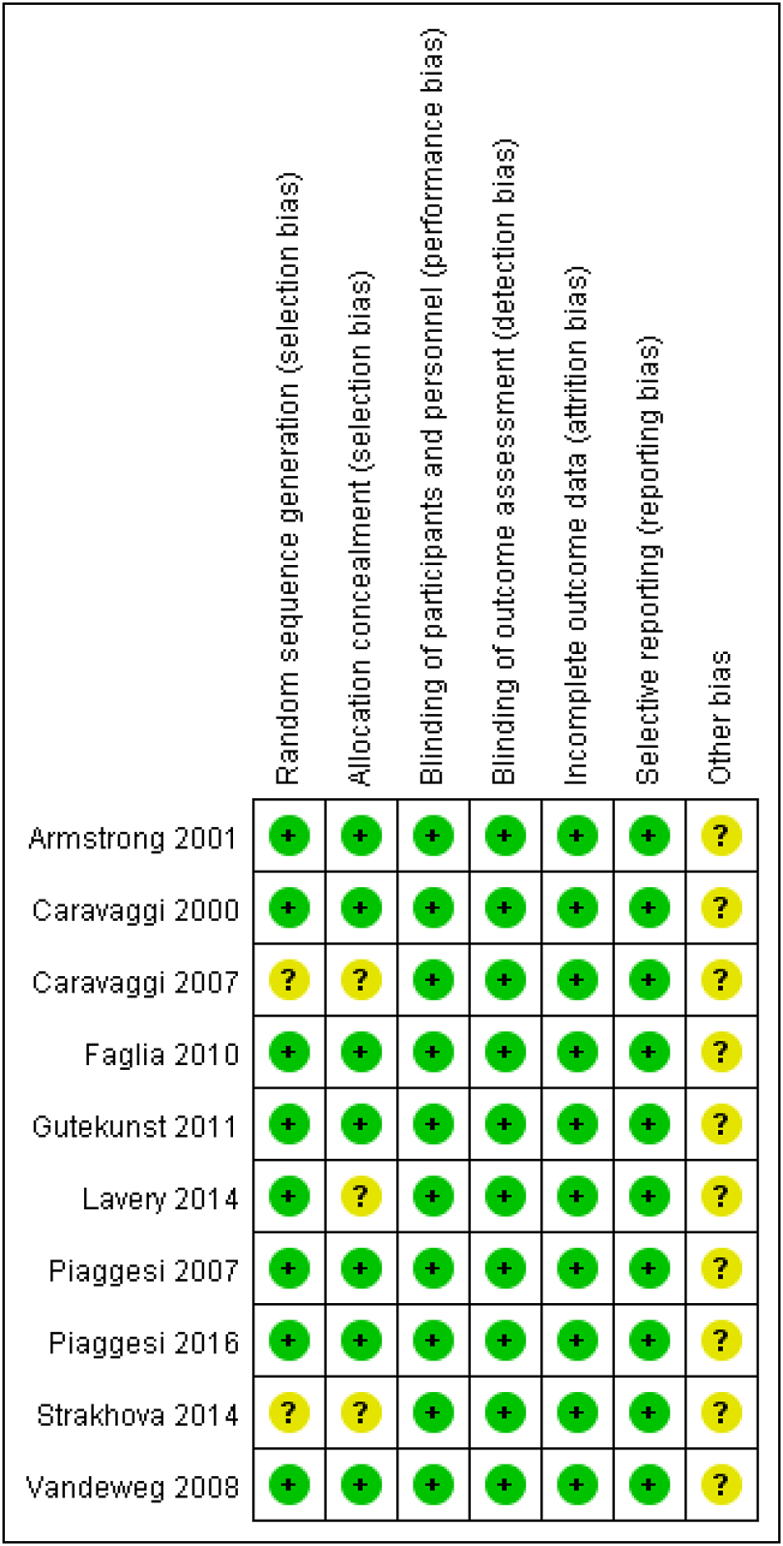
Risk of bias of each RCT.

The quality of clinical trials is summarized in **Figures 2**, **3**, and **Table 2**. None of the included RCTs were rated to have a high risk of bias in terms of incomplete outcome data, blinding of participants and personnel, outcome assessment, and selection of reported results. Specifically, two studies from the same pool were prone to unclear risk of bias regarding random sequence generation and received a low risk of bias score in other methodological domains. Three RCTs were rated to have an unclear risk of bias regarding allocation concealment and low risk of bias in other domains. Additionally, RCTs were pone to an unclear risk of bias in the domains of bias due to other factors. Among non-randomized controlled trials, two were rated to have a high risk of bias in the Comparability category, while they were prone to a low risk of bias in Selection and Outcome categories.

**Table 2:** Quality evaluation of the eligible non-RCT studies with Newcastle-Ottawa scale.

Study	Selection				Comparability		Outcome		
	Representative-ness	Selection of non-exposed	Ascertainment of exposure	Outcome not present at start	Comparability on most important factors	Comparability on other risk factors	Assessment of outcome	Long enough follow-up (median≥3 months)	Adequacy (completeness) of follow-up (<10%)
Van (24)	☆	☆	☆	☆			☆	☆	☆
Birke (25)	☆	☆	☆	☆			☆	☆	☆

The meaning of the symbol ☆ is yes.

### Rates of ulcer healing

**Figure 4:**
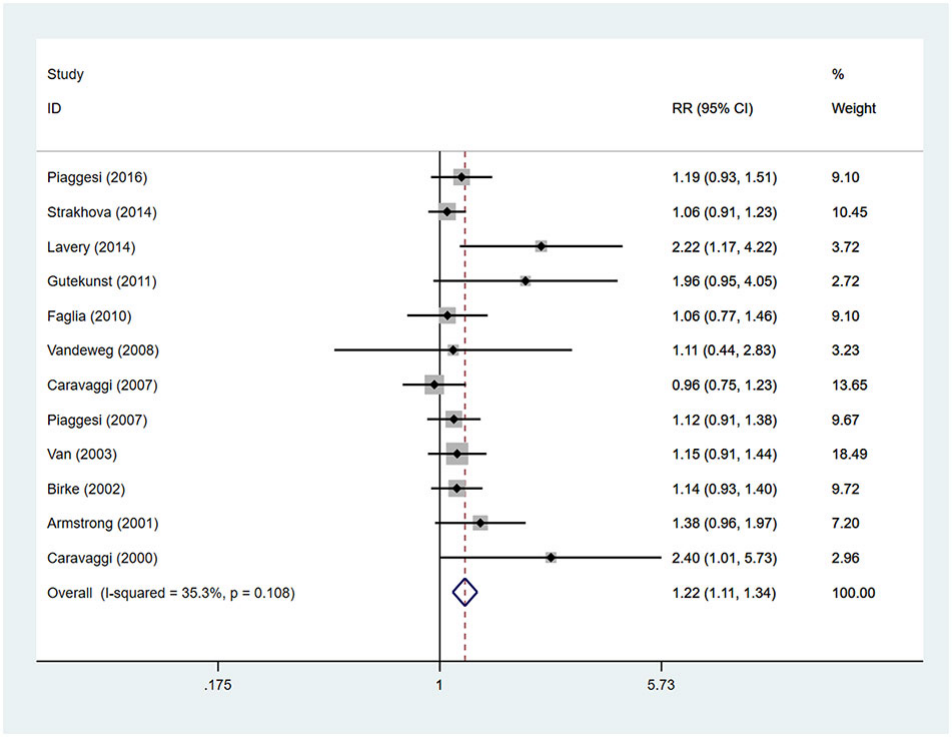
Forest plot of the comparison of the healing rate of TCC and other removable interventions. RR, relative risk.

**Figure 5:**
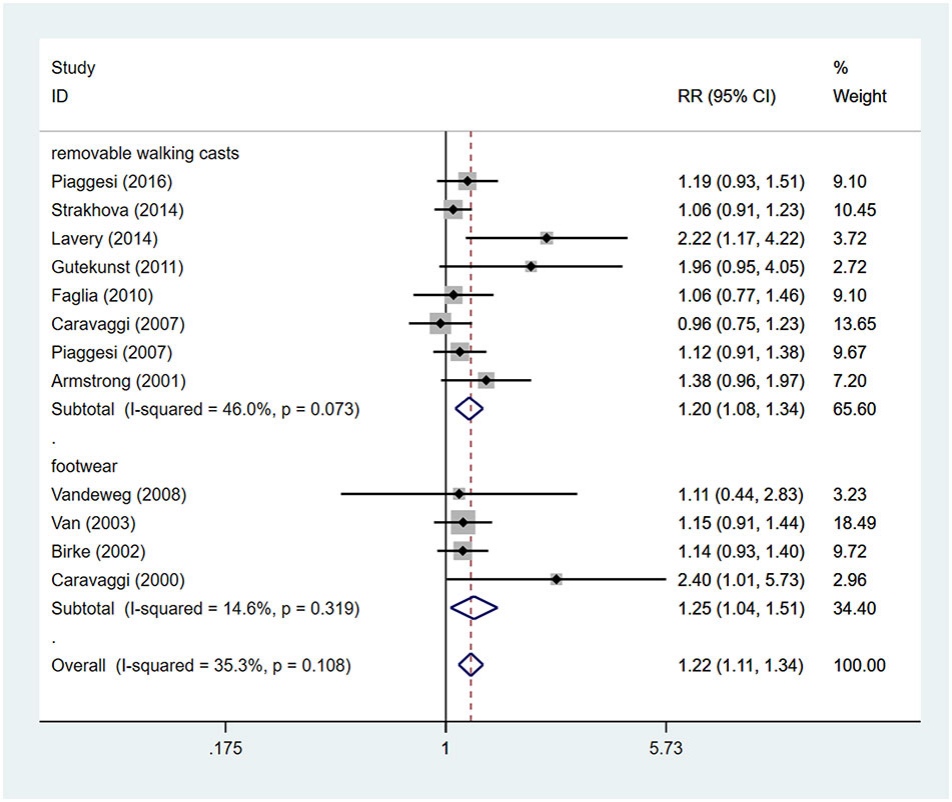
Subgroup analysis of the comparison of the healing rate of TCC and other removable interventions (removable walking casts, footwear). RR, relative risk.

Patients using TCCs had significant higher rates of ulcer healing, compared to those in the control group (RR=1.22; 95% CI: 1.11 to 1.34; p<0.001) (**Figure 4**), with *I^2^* being 35.3% (P=0.108). Subgroup analyses assessing differences in rates of ulcer healing between three types of offloading devices demonstrated very consistent results. Rates of healing were significantly higher in participants using TCCs than in those using removable walking casts (RR=1.20; 95% CI:1.08 to 1.34; p=0.001) or footwear (RR=1.25; 95% CI: 1.04 to 1.51; p=0.019) (**Figure 5**).

### Time for healing

**Figure 6:**
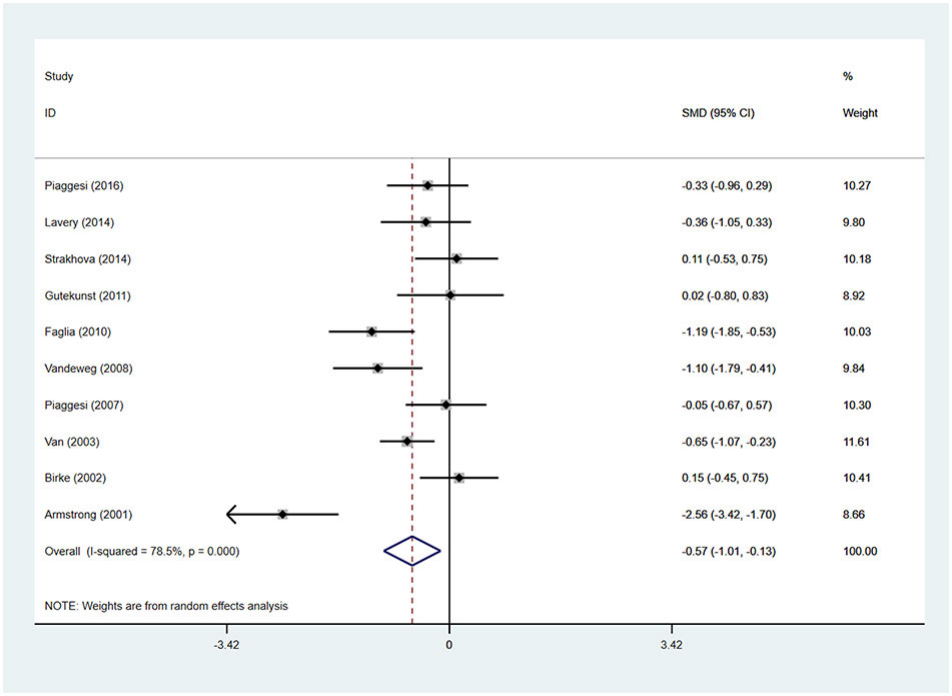
Forest plot of the comparison of the healing time of TCC and other removable interventions. SMD, standard mean difference.

**Figure 7:**
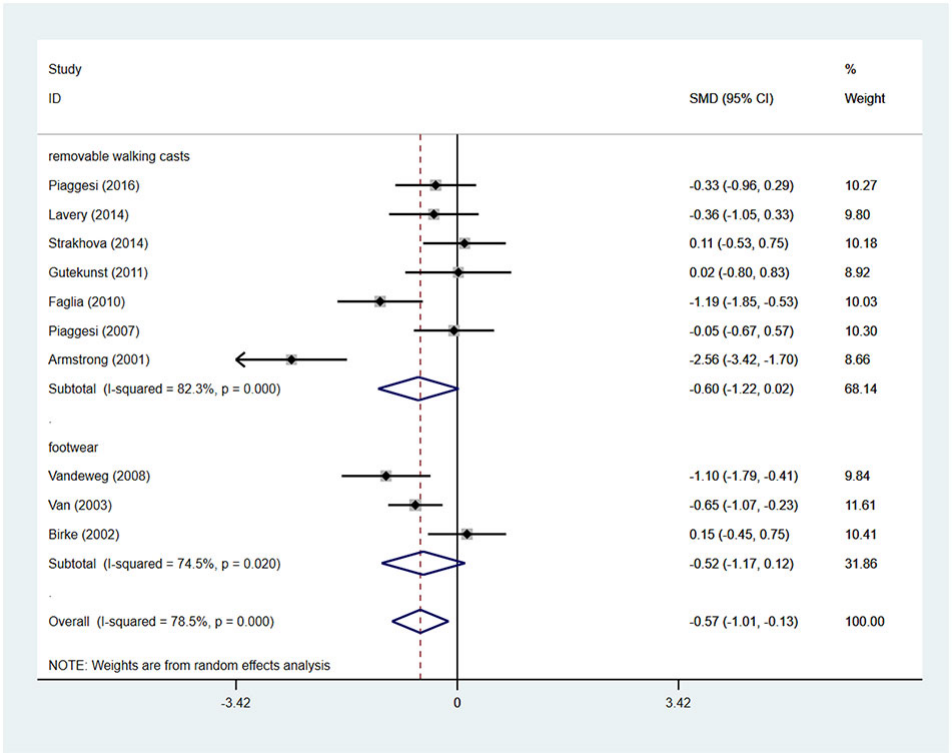
Subgroup analysis of the comparison of the healing time of TCC and other removable interventions (removable walking casts, footwear). RR, relative risk.

Patients using TCCs spent significantly shorter time healing foot ulcers than those using removable walking casts or footwear (SMD=-0.57; 95% CI: −1.01 to −0.13; p=0.010) (**Figure 6**), with *I^2^* being 78.5% (P<0.0001). However, subgroup analyses comparing TCC versus removable walking casts versus footwear showed no significant difference in the time for healing either between TCCs and removable walking casts (SMD=-0.60; 95% CI: −1.22 to 0.02; p=0.058) or between TCCs and footwear (SMD=-0.52; 95% CI: −1.17 to 0.12; p=0.110) (**Figure 7**).

### Device-related complications

**Figure 8:**
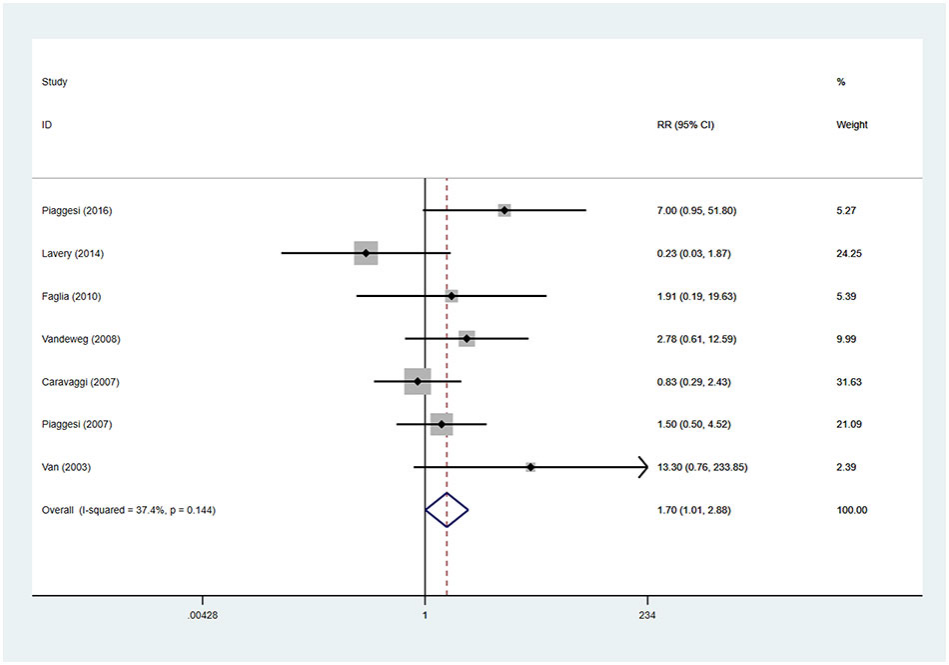
Forest plot of the comparison of the device related complications of TCC and other removable interventions. RR, relative risk.

**Figure 9:**
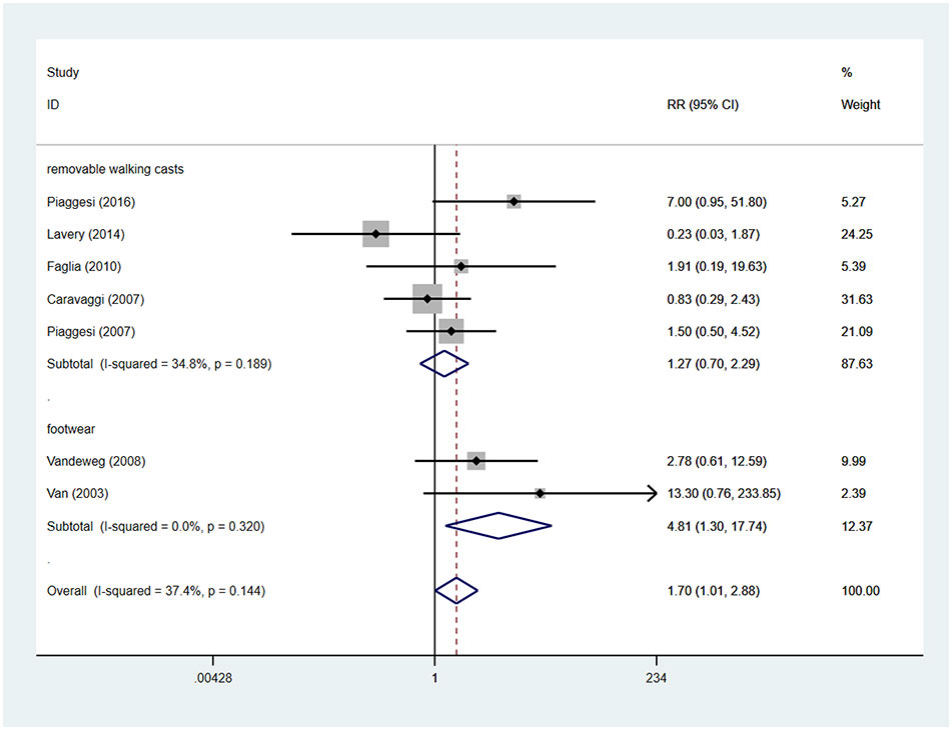
Subgroup analysis of the comparison of the device related complications of TCC and other removable interventions (removable walking casts, footwear). RR, relative risk.

There were seven studies reported the device-related complications. In TCCs group, there were 30 device-related complications, of these complications, 11 were device failures, 13 were skin complications (skin abrasions or skin maceration which can heal on its own), 6 were wound infections. In control group (removable walking casts and footwear), there were 19 device-related complications, of these complications, 11 were wound infections, 6 were skin complications (skin abrasions or skin maceration which can heal on its own), 1 was transient paresthesia with no objective signs, 1 was superficial emathoma of the calf due to accidental trauma. Only 2 complications were reported in footwear subgroup, they were skin abrasions. Detailed information is shown in **Table 3**. Patients using TCCs reported significantly elevated occurrence of device-related complications than those in in the control group (removable walking casts and footwear) (RR=1.70; 95%CI:1.01 to 2.88, P=0.047) (**Figure 8**), with *I^2^* being 37.4% (P=0.144). Subgroup analyses based on three types of devices showed that increased prevalence of device-related complications associated with TTCs was found only in the comparison of TCCs versus footwear (RR=4.81; 95% CI: 1.30 to 17.74; p=0.018), but not in that of TCCs versus removable walking casts (RR=1.27; 95CI: 0.70 to 2.29; p=0.438) (**Figure 9**).

**Table 3:** Device-related complications in studies.

Study	TCC		Control	
	Number	complications	Number	complications
Piaggesi 2016 (12)	7	4 traumatic abrasions 3 device failure	1	1 fungal intertrigo
Lavery 2014	1	1 infection	5	4 infections 1 device-related wounds
Faglia 2010 (13)	2	2 device failure	1	1 skin maceration
Vandeweg 2008 (22)	5	5 device failure	2	2 Minor abrasions
Caravaggi 2007 (23)	5	5 serious infection req-uired antibiotic the-rapy and surgical debridment	6	6 serious infection required antibioti therapy and surgical debrid-ment
Piaggesi 2007 (14)	5	1 device failure 4 skin maceration	4	1 transient paresthesia with no objective signs 2 skin maceration 1 superficial emathoma of the calf due to accidental trauma
Van 2003 (24)	5	5 ulcer caused by the fiberglass	0	
Total	30	11 device failure 13 skin complication 6 wound infections	19	11 wound infection 6 skin complication 2 others

### Sensitivity analysis and publication bias

Sensitivity analyses demonstrated no significance change in the pooled estimates of primary outcomes. Results of Egger’s test demonstrated no publication bias across included studies evaluating the effects of offloading devices regarding the time for ulcer healing (P=0.521) and occurrence of device-related complications (P=0.357). However, publication bias emerged among studies investigating rates of ulcer healing (p=0.007).

## Discussion

Our meta-analysis demonstrated that TCCs significantly reduced healing time and improved healing rates compared to other removable offloading interventions (removable walking casts and footwear). However, TCCs were also associated with increased occurrence of complications. Specifically, we found that the increased prevalence of device-related complications was only observed when TCCs were compared with footwear. This finding has significant implications for the choice of treatment modality in patients with diabetic foot ulcers.

Our findings align with another recent meta-analysis, which also concluded that TCCs outperformed removable walkers because patients using the former spent shorter time on ulcer healing (15). However, that meta-analysis only focused on healing time as the primary outcome and included a limited number of studies (five in total). A systematic review suggested non-removable and knee-high offloading devices (TCCs or non-removable walkers) as the preferred treatment options for plantar neurogenic forefoot and midfoot ulcers (16). Removable offloading devices, whether knee-high or ankle-high, were considered the secondary choice among available offloading interventions. However, it did not include any meta-analyses, so its conclusions were not based on quantitative analysis, nor did it provide information on the incidence of complications associated with individual devices.

Several factors may explain these results. First, there are differences in biomechanical offloading capabilities between TCCs and removable walking casts/footwear. Compared with removable interventions, TCCs can significantly reduce plantar pressure through the shaft effect and load transfer to the contralateral foot (28). Second, patient adherence varies between populations using TCCs versus removable walking casts or footwear.Studies have indicated that consistent use of foot offloading for at least 80% of daily activity is essential for its effectiveness (16, 29). Diabetic foot ulcer healing requires persistent pressure relief. The effect of offloading interventions diminishes when patients remove the device, and persistent abnormal plantar pressure may result in non-healing ulcers. Therefore, the higher adherence observed with TCCs could explain their superior effectiveness in promoting healing-related outcomes compared to removable devices. Our findings underscore the importance of patient education and adherence in managing diabetic foot ulcers. However, not all patients can adhere to these principles when using removable walking casts or footwear. A research had shown that patients, on average, used removable walking casts only for about 59% ( ± 22%) of their total daily activity (30), and therapeutic footwear was used for an average of 50.3% ( ± 32.8%) (31). The variation in comfort among different offloading devices may also influence patient compliance (32).

While patients with DFUs using TCCs demonstrated better healing time and healing rates, the use of TCCs was associated with greater frequency of complications compared to removable offloading interventions (12–14, 22, 24). This could be attributed to the fact that patients using removable offloading interventions have the option to remove the device, which allows them to identify any changes in their foot condition at an early stage.

We identified two main types of complications with TCCs: device failure and skin problems. To address device failure, we propose two strategies. First, improving the quality of the casts used can reduce the likelihood of device failure. Second, educating patients on the importance of protecting their TCCs could also reduce the incidence of this complication. As for skin complications, proper molding of the cast is crucial to avoid local compression and prevent abrasions as well as other skin problems. Furthermore, keeping the patient’s foot dry and clean can help prevent skin maceration and subsequent infection. These strategies, if properly implemented, could potentially enhance the effectiveness of TCCs in treating diabetic foot ulcers.

It is important to acknowledge some limitations of our study (1): Some included trials had a small sample size, with seven studies including less than 50 participants (2). Significant heterogeneity was observed in terms of healing time, prompting us to conduct subgroup analyses to identify and minimize this heterogeneity (3). Each removable walking cast differs in terms of unloading ability and wearing comfort, potentially affecting patient compliance and ulcer healing.

In conclusion, our meta-analysis indicates that TCCs resulted in a shorter time to heal foot ulcers and improved healing rates compared to removable offloading interventions in patients with DFUs. Our study also emphasizes the need for clinicians to consider the potential for increased device-related complications with TCCs, especially when compared with footwear. These findings have potential clinical implications for the selection of appropriate offloading interventions to optimize treatment outcomes and reduce complications. Future research should focus on enhancing the design of offloading devices to enhance patient compliance and minimize complications while maintaining effective offloading.

## Data availability statement

The original contributions presented in the study are included in the article/supplementary material, further inquiries can be directed to the corresponding author.

## Author contributions

Conceptualization: BL, JH. Data curation: BL, JH. Formal analysis: BL, JH. Funding acquisition: BL, JX. Investigation: BL, JX. Methodology: BL, JX. Project administration: AL, QL. Resources: AL, QL. Software: AL, CY. Supervision: AL, CY. Validation: AL, ZZ. Visualization: JH, ZZ. Writing -original draft: BL, AL, JH, JX, QL, CY, ZZ. Writing-review & editing: BL, AL, JH, JX, QL, CY, ZZ. All authors contributed to the article and approved the submitted version.

## Appendix

Pubmed search strategy: Search: ((((((Diabetic foot[Title/Abstract]) OR (Foot, Diabetic[Title/Abstract])) OR (Diabetic Feet[Title/Abstract])) OR (Feet, Diabetic[Title/Abstract])) OR (Foot Ulcer, Diabetic[Title/Abstract])) OR ("Diabetic Foot"[Mesh])) AND (((((((((((((((((((((((Casts, Surgical[Title/Abstract]) OR (Surgical Casts[Title/Abstract])) OR (Cast, Surgical[Title/Abstract])) OR (Surgical Cast[Title/Abstract])) OR (Plastic Casts[Title/Abstract])) OR (Cast, Plastic[Title/Abstract])) OR (Casts, Plastic[Title/Abstract])) OR (Plastic Cast[Title/Abstract])) OR (Plaster Casts[Title/Abstract])) OR (Cast, Plaster[Title/Abstract])) OR (Casts, Plaster[Title/Abstract])) OR (Plaster Cast[Title/Abstract])) OR (Fiberglass Casts[Title/Abstract])) OR (Cast, Fiberglass[Title/Abstract])) OR (Casts, Fiberglass[Title/Abstract])) OR (Fiberglass Cast[Title/Abstract])) OR (total contact casts[Title/Abstract])) OR (total contact casting[Title/Abstract])) OR (total contact cast[Title/Abstract])) OR (cast[Title/Abstract])) OR (casts[Title/Abstract])) OR (casting[Title/Abstract]) AND (clinicaltrial[Filter] OR randomizedcontrolledtrial[Filter])) OR ("Casts, Surgical"[Mesh]) AND (clinicaltrial[Filter] OR randomizedcontrolledtrial[Filter]))

Embase search strategy: ('diabetic foot'/exp OR 'diabetic foot':ab,ti OR 'foot, diabetic':ab,ti OR 'diabetic feet':ab,ti OR 'feet, diabetic':ab,ti OR 'foot ulcer, diabetic':ab,ti) AND ('orthopedic cast'/exp OR 'orthopedic cast':ab,ti OR 'casts, surgical':ab,ti OR 'surgical casts':ab,ti OR 'cast, surgical':ab,ti OR 'surgical cast':ab,ti OR 'plastic casts':ab,ti OR 'cast, plastic':ab,ti OR 'casts, plastic':ab,ti OR 'plastic cast':ab,ti OR 'plaster casts':ab,ti OR 'cast, plaster':ab,ti OR 'casts, plaster':ab,ti OR 'plaster cast':ab,ti OR 'fiberglass casts':ab,ti OR 'cast, fiberglass':ab,ti OR 'casts, fiberglass':ab,ti OR 'fiberglass cast':ab,ti OR 'total contact casts':ab,ti OR 'total contact casting':ab,ti OR 'total contact cast':ab,ti OR 'cast':ab,ti OR 'casts':ab,ti OR 'casting':ab,ti) AND ('case control study'/de OR 'clinical article'/de OR 'clinical trial'/de OR 'cohort analysis'/de OR 'comparative study'/de OR 'controlled clinical trial'/de OR 'controlled study'/de OR 'crossover procedure'/de OR 'double blind procedure'/de OR 'major clinical study'/de OR 'randomized controlled trial'/de OR 'randomized controlled trial topic'/de OR 'single blind procedure'/de) AND ('case control study'/de OR 'clinical article'/de OR 'clinical trial'/de OR 'cohort analysis'/de OR 'comparative study'/de OR 'controlled clinical trial'/de OR 'controlled study'/de OR 'crossover procedure'/de OR 'double blind procedure'/de OR 'major clinical study'/de OR 'prospective study'/de OR 'randomized controlled trial'/de OR 'randomized controlled trial topic'/de OR 'retrospective study'/de OR 'single blind procedure'/de)

Cochrane Library search strategy:

#1 MeSH descriptor: [Diabetic Foot] explode all trees
#2 (Diabetic foot):ti,ab,kw OR (Foot, Diabetic):ti,ab,kw OR (Diabetic Feet):ti,ab,kw OR (Feet, Diabetic):ti,ab,kw OR (Foot Ulcer, Diabetic):ti,ab,kw (Word variations have been searched)
#3 #1 OR #2
#4 MeSH descriptor: [Casts, Surgical] explode all trees
#5 (Casts, Surgical):ti,ab,kw OR (Surgical Casts):ti,ab,kw OR (Cast, Surgical):ti,ab,kw OR (Surgical Cast):ti,ab,kw OR (Plastic Casts):ti,ab,kw (Word variations have been searched)
#6 (Cast, Plastic):ti,ab,kw OR (Casts, Plastic):ti,ab,kw OR (Plastic Cast):ti,ab,kw OR (Plaster Casts):ti,ab,kw OR (Cast, Plaster):ti,ab,kw (Word variations have been searched)
#7 (Casts, Plaster):ti,ab,kw OR (Plaster Cast):ti,ab,kw OR (Fiberglass Casts):ti,ab,kw OR (Cast, Fiberglass):ti,ab,kw OR (Casts, Fiberglass):ti,ab,kw (Word variations have been searched)
#8 (Fiberglass Cast):ti,ab,kw OR (total contact casts):ti,ab,kw OR (total contact casting):ti,ab,kw OR (total contact cast):ti,ab,kw OR (cast):ti,ab,kw (Word variations have been searched)
#9 (casts):ti,ab,kw OR (casting):ti,ab,kw (Word variations have been searched)
#10 #4 OR #5 OR #6 OR #7 OR #8 OR #9
#11 #3 AND #10
